# Post-Intoxication Inhibition of Botulinum Neurotoxin Serotype A within Neurons by Small-Molecule, Non-Peptidic Inhibitors

**DOI:** 10.3390/toxins3030207

**Published:** 2011-03-15

**Authors:** Gordon Ruthel, James C. Burnett, Jonathan E. Nuss, Laura M. Wanner, Lyal E. Tressler, Edna Torres-Melendez, Sarah J. Sandwick, Cary J. Retterer, Sina Bavari

**Affiliations:** 1 U.S. Army Medical Research Institute of Infectious Diseases, 1425 Porter Street, Frederick, MD 21702; USA; Email: jonathan.nuss@amedd.army.mil (J.E.N.); laura.wanner@amedd.army.mil (L.M.W.); lyal.tressler@amedd.army.mil (L.E.T.); edna.torres-melendez@amedd.army.mil (E.T.-M.); sarah.sandwick@uni-wuerzburg.de (S.J.S.); cary.retterer@amedd.army.mil (C.J.R.); 2 SAIC-Frederick, Inc., National Cancer Institute at Frederick, Target Structure-Based Drug Discovery Group, P.O. Box B, Frederick, MD 21702, USA; Email: burnettjames@mail.nih.gov

**Keywords:** botulinum neurotoxin, small molecule non-peptidic inhibitor, neutralizing antibody, Bafilomycin A1

## Abstract

Botulinum neurotoxins (BoNTs) comprise seven distinct serotypes that inhibit the release of neurotransmitter across neuromuscular junctions, resulting in potentially fatal flaccid paralysis. BoNT serotype A (BoNT/A), which targets synaptosomal-associated protein of 25kDa (SNAP-25), is particularly long-lived within neurons and requires a longer time for recovery of neuromuscular function. There are currently no treatments available to counteract BoNT/A after it has entered the neuronal cytosol. In this study, we examined the ability of small molecule non-peptidic inhibitors (SMNPIs) to prevent SNAP-25 cleavage post-intoxication of neurons. The progressive cleavage of SNAP-25 observed over 5 h following 1 h BoNT/A intoxication was prevented by addition of SMNPIs. In contrast, anti-BoNT/A neutralizing antibodies that strongly inhibited SNAP-25 cleavage when added during intoxication were completely ineffective when added post-intoxication. Although Bafilomycin A1, which blocks entry of BoNT/A into the cytosol by preventing endosomal acidification, inhibited SNAP-25 cleavage post-intoxication, the degree of inhibition was significantly reduced versus addition both during and after intoxication. Post-intoxication application of SMNPIs, on the other hand, was nearly as effective as application both during and after intoxication. Taken together, the results indicate that competitive SMNPIs of BoNT/A light chain can be effective within neurons post-intoxication.

## 1. Introduction

Botulinum neurotoxins (BoNTs) are the most potent of known biological toxins [1]. By proteolytically cleaving components of the soluble *N*-ethylmaleimide-sensitive factor attachment protein receptor (SNARE) complex, BoNTs impair neuronal synaptic vesicle fusion, thus preventing neurotransmission across neuromuscular junctions [2]. This leads to the flaccid paralysis associated with the disease state botulism. Of key importance, once diaphragm and external intercostal muscles are paralyzed due to BoNT poisoning, death by suffocation results unless critical care intervention is implemented [3]. BoNTs are listed as category A biothreat agents by the Centers for Disease Control and Prevention, with the potential to be delivered by various routes [1,4], which could result in a large-scale outbreak that would overwhelm our current health care system.

There are seven known BoNT serotypes (designated A–G) [2]. All possess a zinc metalloprotease component, referred to as the light chain (LC), with each serotype targeting a different and specific peptide bond in one or more of three SNARE proteins. BoNT serotypes A, C, and E cleave synaptosome-associated protein of 25 kDa (SNAP-25). BoNT serotype C also cleaves syntaxin. BoNT serotypes B, D, F, and G cleave synaptobrevin [2]. Of the seven serotypes, BoNT serotype A (BoNT/A) persists the longest within neurons [5] and likewise requires the longest time for recovery of neuromuscular function. This long duration of activity has made BoNT/A the serotype of choice for many medical applications (such as the treatment of strabismus, cervical dystonia, and blepharospasm) [6,7], as well as the cosmetic reduction of facial wrinkles [8]. Consequently, the clinical value of BoNT/A has made vaccination a less desirable option for preventing botulism, as vaccination would neutralize the potential therapeutic benefits of the enzyme. However, the increasing use of BoNT/A in clinical applications also carries with it a heightened risk of accidental overdose. In addition, intentional misuse of BoNT/A for bioterrorism remains a concern. For these reasons, the discovery and development of therapeutics to counter BoNT/A intoxication is of particular importance. The current treatment for BoNT intoxication (botulism) consists of anti-toxin therapy [3]. Specifically, antibodies are administered that bind to BoNT and aid in clearing it from the bloodstream [3], thereby preventing further entry of the toxin into neurons. However, once BoNT/A (or any BoNT) has entered the neuronal cytosol, it is inaccessible to these antibodies, rendering antitoxin treatment ineffective against the persistent cleavage of SNARE proteins [9]. In severe cases, the only recourse after BoNT-induced paralysis manifests is mechanical ventilation to keep individuals with botulism alive until recovery, which can take several months. In a documented case of cosmetic injection with concentrated and unlicensed BoNT/A, one patient required nearly 6 months of ventilator support to survive [10]. This patient did not receive antitoxin until more than a week after exposure to the toxin. A second patient, who was injected with the same unlicensed BoNT/A and had a similar serum toxin level, received antitoxin 3 days after exposure and, in contrast, was able to be taken off ventilator support after little more than a month [10]. Although this example demonstrates the efficacy of the antitoxin when administered soon after intoxication, it also demonstrates that the window of opportunity for effective treatment with antitoxin is relatively narrow. Hence, the search for small-molecule non-peptidic inhibitors (SMNPIs) of BoNT/A has been driven by the hypothesis that such inhibitors, unlike anti-BoNT antibodies, will possess the ability to counter BoNT/A LC enzymatic activity within the neuronal cytosol.

While cell-free biochemical assays are useful for the initial selection of compounds that inhibit BoNT/A LC enzymatic activity, potent inhibition in these assays does not guarantee effectiveness in the neuronal cytosol. In contrast, cell-based assays can be used to test the post-intoxication intracellular efficacy of inhibitors of BoNT/A by intoxicating cells and rinsing away the remaining extracellular toxin before adding SMNPI. In this study, this assay was used to demonstrate the ability of two SMNPIs, NSC 95654 and NSC 104999, to prevent BoNT/A induced cleavage of SNAP-25 after intoxication of neurons.

## 2. Materials and Methods

### 2.1. Neuronal Cell Culture

Ventral spinal cords were isolated from day 6 embryonic chicken embryos (Charles River Laboratories, North Franklin, CT) and cultured by the method of Kuhn [11] as previously described [12]. Briefly, spinal cords were removed from the embryos and the dorsal halves trimmed away. After trypsinization for 25 min, the cells were dissociated by trituration and pre-plated in Dulbecco’s modified Eagle’s medium for 1 h to enrich the neuronal population by adherence of non-neuronal cells to the culture dish. Cells remaining in suspension were centrifuged for 6 min at 1250 rpm, resuspended in Liebowitz’s L15 medium with 10% fetal bovine serum and N3 supplements, and plated onto 6-well culture plates that were first coated with poly-L-lysine (1 mg/mL overnight), and then with laminin (7–10 μg/mL for 3–4 h). Neurons were incubated overnight at 37 °C in 5% CO_2_ before use in the experiments.

### 2.2. BoNT/A and Antagonists

BoNT/A was purchased from Metabiologics Inc. (Madison, WI). NSC 95654 and NSC 104999 were obtained from the National Cancer Institute’s Open Repository. Stock solutions of these compounds were prepared at 10 µM in DMSO and diluted to final concentrations as indicated in cell culture medium. Anti-BoNT/A neutralizing antibodies (6E10-10 and 4A2-4) were produced in the U.S. Army Medical Research Institute of Infectious Diseases [13] and were previously shown to be highly effective inhibitors of BoNT/A when applied together with the toxin to chick motor neuron cultures [12]. Results with each of the two antibodies were similar and were thus pooled for purposes of graphing and statistical analysis. Bafilomycin A1 was purchased from Tocris bioscience (Ellisville, MO).

### 2.3. Western Blot Analysis

Cells were gently scraped and collected into tubes. The tubes were centrifuged, then supernatant was removed and replaced with 30 µL of lysis buffer. After overnight storage at −80 °C, tubes were thawed, vortexed briefly, and incubated on ice for 10 min. Lysates were centrifuged at high speed at 4 °C for 30 min to remove debris. Total protein was measured by the Bradford assay. 12% gels were used for SDS PAGE and proteins were blotted onto PVDF membranes. Western blots were probed with a 1:2500 dilution of SMI-81 anti-SNAP-25 antibody (Covance Research Products Inc., Berkeley, CA) and 1:20,000 horseradish peroxidase (HRP)-conjugated goat anti-mouse (Pierce, Rockford, IL). Western blots were developed using a chemiluminescent substrate and imaged with a BioRad Versadoc system. Protein bands were quantitated by densitometry with Quantity One software (BioRad, Hercules, CA). 

### 2.4. *In Vitro* Evaluation of Small-Molecule Inhibitors

Inhibition of BoNT/A LC metalloprotease activity by NSC 95654 and NSC 104999 *in vitro* was measured employing an HPLC-based assay developed by Schmidt and Bostian [14]. In brief , a synthetic *N*-terminal acetylated, *C*-terminal aminated peptide (New England Peptide, Gardner, MA) corresponding to residues 187–203 of SNAP-25 was used as the substrate for recombinant BoNT/A LC (List Laboratories, Campbell, CA). Assays were conducted by diluting peptide substrate (100 µM) and SMNPIs (1–30 µM) in a buffered solution (40 mM HEPES (pH 7.3), 0.05% Tween 20, 0.5 mg/mL bovine serum albumin, 1 mM dithiothreitol, and 50 µM ZnCl_2_) and initiating reactions by adding BoNT/A LC. After 10 min, the reactions were quenched by adding trifluoroacetic acid, and analyzed by reverse-phase HPLC. All HPLC measurements were conducted on a Dionex Summit (Sunnyvale, CA) HPLC system equipped with a dual wavelength UV/Vis absorbance detector and used a c18 reverse phase column (3 μm, 10 cm × 2.1 mm) obtained from Supelco/Sigma-Aldrich (St. Louis, MO). IC_50_ values were determined by measuring BoNT/A LC activity at nine different SMNPI concentrations, as well as in the absence of SMNPI. The SMNPI concentrations in these measurements were determined by estimating the IC_50_ value and moving in 0.5 log increments in either direction of the estimated value. The resulting kinetic data were then fit to the Langmuir isotherm, *V_i_*/*V_o_* = 1/1 + ([I]/IC_50_)^h^, using nonlinear regression analysis, to obtain *K_i_* values. All reported values are averages of at least four independent experiments.

## 3. Results and Discussion

**Figure 1:**
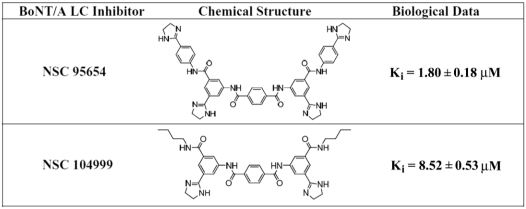
Chemical structures and *K_i_* values for NSC 95654 and NSC 104999.

Previous research [15] led to the identification of NSC 104999, a terephthalamide-based SMNPI of the BoNT/A LC metalloprotease (Figure 1). As part of the current study, various analogs of this SMNPI chemotype were obtained and examined for *in vitro* potency employing an HPLC-based assay. Of the examined analogs, NSC 95654 (Figure 1), was found to be substantially more potent *in vitro* (*K_i_* = 1.80 ± 0.18 µM) than either NSC 104999 (*K_i_* = 8.52 ± 0.53 µM) or the previously reported [16] BoNT/A LC inhibitor NSC 240898 (*K_i_* = 10.5 ± 1.10 µM). The higher potency of NSC 95654 suggests that the synthetic modification of terephthalamide-based SMNPIs can be used to increase the inhibitory potency of this chemotype. Like NSC 240898, NSCs 95654 and 104999 are competitive inhibitors that do not act via Zinc (Zn^++^) chelation, as increasing concentrations of Zn^++^ (from 5 to 50 µM) had no effect on the ability of the SMNPIs to inhibit BoNT/A LC activity in an *in vitro*, HPLC-based assay (data not shown).

**Figure 2:**
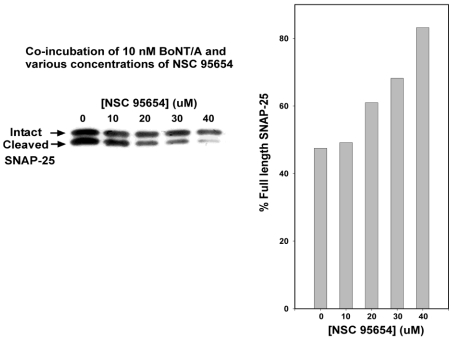
Inhibition of BoNT/A-mediated SNAP-25 cleavage during co-incubation with various concentrations of NSC 95654. Embryonic chick spinal motor neurons were incubated for 3 h in 10 nM BoNT/A and 0–40 µM NSC 95654. Western blotting was performed to reveal amounts of cleaved and intact SNAP-25. Band densities were quantitated and are represented graphically as the percentage of intact SNAP-25.

**Figure 3:**
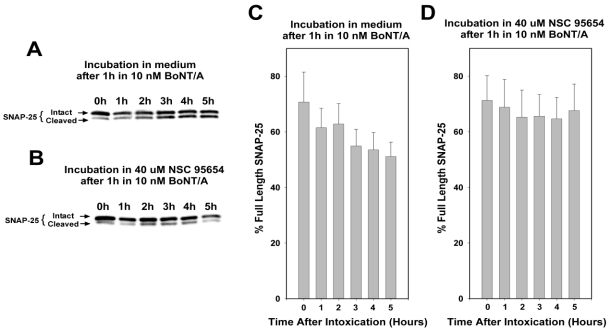
Progressive SNAP-25 cleavage in neurons post-intoxication. Embryonic chick motor neuron cultures were incubated for 1 h in 10 nM BoNT/A, and then residual BoNT/A was removed by rinsing the cells three times with medium. Finally, the cells were collected for Western blot analysis at 1, 2, 3, 4, and 5 h after removal of extracellular (*i.e.*, residual) BoNT/A. (**A**) In the presence of medium alone, there was progressively more cleaved SNAP-25 (lower band) relative to intact SNAP-25 (upper band); (**B**) When NSC 95654 was added to neurons after the removal of residual BoNT/A, little or no change was apparent in the relative intensities of the bands corresponding to intact and cleaved SNAP-25. Graphs based on densitometry readings of Western blot bands show the means and standard deviations for repeat experiments (*N* = 4) for post-intoxication incubation in (**C**) medium alone or (**D**) 40 uM NSC 95654. By 5 h after removal of residual BoNT/A by rinsing, a significantly lower percentage of SNAP-25 remained intact (*P* = 0.017, *t*-test, compared to 0 h) when the neurons were incubated in medium alone versus when incubated with 40 uM NSC 95654 (*P* = 0.595, *t*-test, compared to 0 h).

Consistent with *in vitro* results, a preliminary analysis in which chick spinal motor neurons were incubated for 3 h with 10 nM BoNT/A showed substantial and dose-dependent protection against SNAP-25 cleavage when co-incubated with NSC 95654 (Figure 2). These preliminary results indicated that NSC 95654 was much more effective (approximately twofold) at inhibiting SNAP-25 cleavage in a cell-based assay than the previously reported NSC 240898 [16]. However, co-incubation of cells with BoNT/A and SMNPI does not demonstrate conclusively that the enzyme is being inhibited post-intoxication (*i.e.*, after it has already entered the neuronal cytosol). Because an SMNPI would be of greatest clinical value as a therapeutic if it is able to inhibit, within the neuronal cytosol, the ongoing cleavage of SNARE proteins by BoNT (*i.e.*, to complement anti-toxin treatment, which helps clear BoNT from the bloodstream), we performed detailed studies to determine if NSC 95654 could prevent BoNT/A mediated SNAP-25 cleavage when administered to neurons post-intoxication. To examine the ability of NSC 95654 to protect SNAP-25 in neurons post-intoxication, we first determined the experimental parameters necessary to detect the on-going cleavage of SNAP-25 that occurs after BoNT/A has been washed from the surrounding medium. Casual observation suggested that NSC 95654 was well tolerated by cells over a period of several hours, but that some cell death occurred after overnight incubation. Therefore, we wished to minimize cytotoxicity by minimizing post-intoxication incubation time. To this end, embryonic chick spinal motor neurons were incubated with 10 nM BoNT/A for 1 h, and then washed three times with fresh medium. Cells were collected for Western blot analysis of intact and cleaved SNAP-25 either shortly after the removal of BoNT/A or at 1 h intervals up to 5 h. As expected based on the previously reported persistence of BoNT/A LC protease activity within cells [9], we observed progressive cleavage of SNAP-25 after residual BoNT/A had been washed from the medium surrounding the cells (Figure 3, A and C). One way analysis of variance (ANOVA) revealed significant differences (*P* = 0.014) in SNAP-25 cleavage over time. The degree of SNAP-25 cleavage was statistically significant by 4 and 5 h after removal of BoNT/A (*P* = 0.039 and *P* = 0.015, respectively; pairwise comparison with the 0 h timepoint by Tukey Test). In contrast, when 40 µM NSC 95654 was added to the cells immediately after residual BoNT/A was fully rinsed away, no statistically significant additional SNAP-25 cleavage was detected (*P* = 0.894, one way ANOVA) over the course of 5 h (Figure 3B,D). Comparison of percentage intact SNAP-25 in the absence versus presence of NSC 95654 at 5 h post-intoxication demonstrated a statistically significant difference (*P* = 0.023; *t*-test), further indicating that 5 h was sufficient to detect post-intoxication inhibition. Based on these results, subsequent experiments were performed by intoxicating cells with BoNT/A for 1 h and collecting cells for Western blot analysis 5h after residual BoNT/A was thoroughly rinsed from the culture medium.

**Figure 4:**
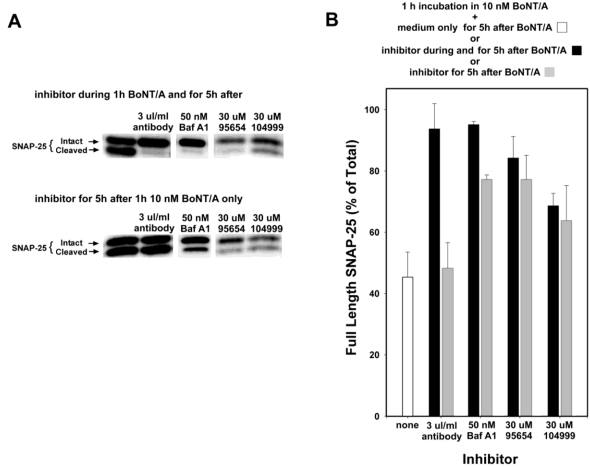
NSC 95654 and NSC 104999, but not BoNT/A neutralizing antibodies, inhibit the cleavage of SNAP-25 by internalized BoNT/A. Neurons were intoxicated with 10 nM BoNT/A for 1 h, rinsed thoroughly, and collected 5 h later for Western blot analysis. (**A**) When BoNT/A neutralizing antibodies, Bafilomicin A1, NSC 95654, or NSC 104999 were added to neuronal cultures during, as well as after, incubation with BoNT/A, all demonstrated potent inhibition of BoNT/A LC-mediated SNAP-25 cleavage. However, neutralizing antibodies failed to protect BoNT/A-mediated SNAP-25 cleavage when added only after non-internalized BoNT/A was removed. In contrast, Bafilomycin A1, NSC 95654, and NSC 104999 remained effective when applied post-intoxication; (**B**) Band densities were determined from the Western blot results and graphed as means and standard deviations from multiple experiments (*N* ≥ 4). Inhibitor treatments resulted in a significantly higher percentage of intact SNAP-25 (*P* < 0.001, t-test) versus when cells were intoxicated but untreated, except when neutralizing antibodies were applied only after intoxication (*P* = 0.500, *t*-test).

Anti-BoNT/A antibodies do not enter cells, but instead inhibit SNAP-25 cleavage by preventing the entrance of BoNT/A into neurons, and so would not be expected to provide any efficacy if added post-intoxication. Therefore, to confirm that the observed inhibition mediated by NSC 95654 post-intoxication was indeed occurring intracellularly, anti-BoNT/A neutralizing antibodies were tested in the post-intoxication assay. Whereas the simultaneous addition of neutralizing antibody and BoNT/A almost completely prevented the cleavage of SNAP-25, adding the antibody post-BoNT/A intoxication resulted in no detectable inhibition of SNAP-25 cleavage (Figure 4). By comparison, 30 µM NSC 95654 and NSC 104999 inhibited SNAP-25 cleavage regardless of whether the SMNPIs were added to the cells at the same time as BoNT/A, or only after the removal of extracellular toxin (*i.e.*, post-intoxication) (Figure 4). Consistent with SMNPI potencies determined *in vitro* in the HPLC assay (Figure 1), NSC 95654 was more efficacious, with regard to inhibiting BoNT/A LC-mediated SNAP-25 cleavage in the neuronal cytosol, than NSC 104999. 

**Figure 5:**
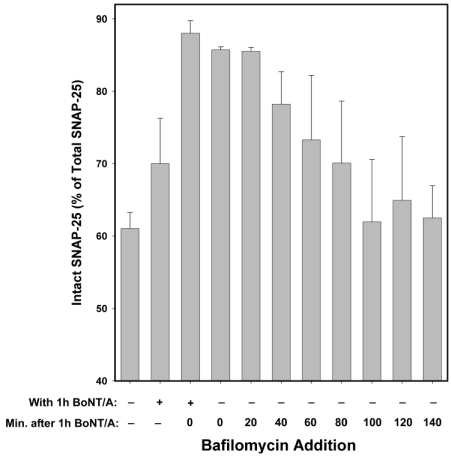
Inhibitory effects of Bafilomycin A1 at different times following 20 min exposure of chick motor neuron cultures to 10 nM BoNT/A. Cells were collected for Western blot analysis 5 h after BoNT/A was removed. Maximal inhibition was observed when Bafilomycin A1 was present throughout the experiment, with hardly any difference if added immediately post-intoxication or at 20 min post-intoxication. The protective efficacy of Bafilomycin A1, with respect to SNAP-25 cleavage, decreased beginning at 40 min post-intoxication. No protection was detected after 100 min post-intoxication. These data suggest that it takes a minimum of 20 min and maximum of 100 min until the BoNT/A LC translocates from the endosome to the cytosol. Because Bafilomycin A1 is a reversible inhibitor of vacuolar-type H^+^-ATPase, the decrease in SNAP-25 cleavage observed when Bafilomycin was present only during intoxication is likely due to a delay in BoNT/A LC cytosol entry. Medians and standard errors were calculated based on a minimum of 6 experiments.

Bafilomycin A1 acts as an inhibitor by preventing the endosomal acidification necessary for translocation of BoNT/A from endosomes into the cytosol. Hence, it acts at a relatively early step in the intoxication process, but at a later step than anti-BoNT/A neutralizing antibodies (which block the binding of BoNT/A to neurons, and hence prevent entry of the toxin). Experiments in which Bafilomycin A1 was added at 20 min intervals, following 20 min intoxication with 10 nM BoNT/A (Figure 5) suggest that the majority of BoNT/A LC translocates from endosomes into the neuronal cytosol approximately 30–90 min after toxin exposure. Therefore, some BoNT/A LC would be expected to have entered the neuronal cytosol after 1 h incubation with BoNT/A. Consequently, BoNT/A LC in the cytosol prior to Bafilomycin A1 addition would be expected to cleave SNAP-25 throughout the remaining 5 h experimental period. Consistent with this hypothesis, the prevention of SNAP-25 cleavage observed when Bafilomycin A1 was present both during and after 1 h BoNT/A intoxication was significantly decreased when Bafilomycin A1 was added post-intoxication (*P* < 0.001, *t*-test; Figure 4). In contrast, NSC 95654 and NSC 104999 are competitive inhibitors of the BoNT/A LC, and consequently can inhibit the enzyme’s catalytic activity regardless of how long after intoxication they are applied. Although there was a small decrease in inhibition of SNAP-25 cleavage when either NSC 95654 or NSC 104999 were added post-intoxication versus when the SMNPIs were also present during intoxication (Figure 4), the difference was not statistically significant (*P* = 0.109 and *P* = 0.346 respectively, *t*-test).

## 4. Conclusions

The ability to enter neurons and inhibit BoNT/A post-intoxication is imperative for any SMNPI of BoNT/A LC enzymatic activity if it is to be an effective treatment for the paralysis resulting from toxin-mediated SNARE protein cleavage. By administering SMNPIs after exposing embryonic chick motor neurons to BoNT/A for 1 h, the results from this study demonstrate that NSC 95654 and NSC 104999 possess the ability to inhibit BoNT/A post-intoxication within the neuronal cytosol. The demonstration of this attribute reinforces the hypothesis that SMNPIs can potentially be employed as therapeutics to treat botulism, and will complement antitoxin administration by inhibiting BoNT/A that is inaccessible to antibodies (*i.e.*, BoNT/A in the neuronal cytosol). Indeed, the post-intoxication efficacies of NSC 95654 and NSC 104999 indicate that these SMNPIs represent a promising chemotype for therapeutic development. Although methods have been developed to test the ability of inhibitors to reduce time to recovery after local injection of BoNT *in vivo* [17], the paradigm for testing post-intoxication efficacy in cell culture that we have presented here is a relatively simple way of confirming intracellular, post-intoxication, efficacy of inhibitors prior to testing in animals.

## References

[1] Paddle B.M. (2003). Therapy and prophylaxis of inhaled biological toxins. J. Appl. Toxicol..

[2] Simpson L.L. (2004). Identification of the major steps in botulinum toxin action. Annu. Rev. Pharmacol. Toxicol..

[3] Arnon S.S., Schechter R., Inglesby T.V., Henderson D.A., Bartlett J.G., Ascher M.S., Eitzen E., Fine A.D., Hauer J., Layton M., Lillibridge S., Osterholm M.T., O’Toole T., Parker G., Perl T.M., Russell P.K., Swerdlow D.L., Tonat K. (2001). Botulinum toxin as a biological weapon: Medical and public health management. JAMA.

[4] Wein L.M., Liu Y. (2005). Analyzing a bioterror attack on the food supply: The case of botulinum toxin in milk. Proc. Nat. Acad. Sci..

[5] Foran P.G., Mohammed N., Lisk G.O., Nagwaney S., Lawrence G.W., Johnson E., Smith L., Aoki K.R., Dolly J.O. (2003). Evaluation of the therapeutic usefulness of botulinum neurotoxin B, C1, E, and F compared with the long lasting type A. J. Biol. Chem..

[6] Comella C.L., Pullman S.L. (2004). Botulinum toxins in neurological disease. Muscle Nerve.

[7] de Maio M. (2008). Therapeutic uses of botulinum toxin: From facial palsy to autonomic disorders. Expert Opin. Biol. Ther..

[8] Weber B.B. (2008). Core curriculum for plastic surgical nursing: Botulinum toxin type A for facial aesthetics. Plast. Surg. Nurs..

[9] O’Sullivan G.A., Mohammed N., Foran P.G., Lawrence G.W., Dolly J.O. (1999). Rescue of exocytosis in botulinum toxin A-poisoned chromaffin cells by expression of cleavage-resistant SNAP-25. J. Biol. Chem..

[10] Chertow D.S., Tan E.T., Maslanka S.E., Schulte J., Bresnitz E.A., Weisman R.S., Bernstein J., Marcus S.M., Kumar S., Malecki J., Sobel J., Braden C.R. (2006). Botulism in 4 adults following cosmetic injections with an unlicensed, highly concentrated botulinum preparation. JAMA.

[11] Kuhn T.B. (2003). Growing and working with spinal motor neurons. Methods Cell Biol..

[12] Stahl A.M., Ruthel G., Torres-Melendez E., Kenny T.A., Panchal R.G., Bavari S. (2007). Primary cultures of embryonic chicken neurons for sensitive cell-based assay of Botulinum neurotoxin: Implications for therapeutic discovery. J. Biomol. Screen..

[13] Pless D.D., Torres E.R., Reinke E.K., Bavari S. (2001). High-affinity, protective antibodies to the binding domain of botulinum neurotoxin type A. Infect. Immun..

[14] Schmidt J.J., Bostian K.A. (1997). Endoproteinase activity of type A botulinum neurotoxin: Substrate requirements and activation by serum albumin. J. Protein Chem..

[15] Hermone A.R., Burnett J.C., Nuss J.E., Tressler L.E., Nguyen T.L., Solaja B.A., Vennerstrom J.L., Schmidt J.J., Wipf P., Bavari S., Gussio R. (2008). Three-dimensional database mining identifies a unique chemotype that unites structurally diverse botulinum neurotoxin serotype A inhibitors in a three-zone pharmacophore. ChemMedChem.

[16] Burnett J.C., Ruthel G., Stegmann C.M., Panchal R.G., Nguyen T.L., Hermone A.R., Stafford R.G., Lane D.J., Kenny T.A., McGrath C.F., Wipf P., Stahl A.M., Schmidt J.J., Gussio R., Brunger A.T., Bavari S. (2007). Inhibition of metalloprotease botulinum serotype A from a pseudo-peptide binding mode to a small molecule that is active in primary neurons. J. Biol. Chem..

[17] Keller J.E. (2006). Recovery from Botulinum neurotoxin poisoning *in vivo*. Neuroscience.

